# Early Dissecting Cellulitis of the Scalp Following Repeated Microinfusion of Medication Into the Skin in a Patient With Androgenetic Alopecia and Long-Term Exogenous Testosterone Use: A Case Report

**DOI:** 10.7759/cureus.108586

**Published:** 2026-05-10

**Authors:** Gabriella A Oliveira, Leonardo De Medeiros Quirino, Cíntia Naomi Kumazawa, Tathiane Oliveira

**Affiliations:** 1 Hair Restoration Surgery, Helio Angotti Hospital, Uberaba, BRA; 2 Medicine, Federal University of Uberlândia, Uberlândia, BRA; 3 Dermatology, Faculdades BWS, São Paulo, BRA; 4 Medical Nutrition and Trichology, Faculty of Medical Sciences, São José dos Campos, São José, BRA

**Keywords:** androgenetic alopecia, cicatricial alopecia, dissecting cellulitis, dissecting folliculitis, exogenous testosterone, microinfusion of medication into the skin, microneedling, trichoscopy

## Abstract

Dissecting cellulitis of the scalp is an uncommon chronic inflammatory disorder within the follicular occlusion spectrum and may progress to permanent cicatricial alopecia if not recognized early. We report a case of a 34-year-old male with androgenetic alopecia, two years of exogenous testosterone use, and a recent history of six-monthly sessions of scalp microinfusion of medication into the skin, who developed painful and pruritic inflammatory scalp nodules approximately 45 days after the last procedure. The procedure was performed using a Cheyenne Unlimited 4 dermograph fitted with a 2710MG cartridge, corresponding to a 27-needle magnum configuration with 0.30 mm diameter needles, operating at 6.1 V, with the needle depth set to 1.2 mm. Clinical examination showed focal inflammatory alopecic nodules over a background of patterned hair thinning. Trichoscopy demonstrated perifollicular erythema, reduced follicular density, broken hairs, black dots, white structureless areas, and partial preservation of follicular openings, without classic features of alopecia areata. Histopathology showed hypodermal granulation tissue, intense mixed leukocytic inflammation composed of lymphocytes, plasma cells, neutrophils, macrophages, and foreign body-type multinucleated giant cells, follicular involvement with miniaturization, negative periodic acid-Schiff staining for fungi, and bacterial colonization in the infundibular and isthmic portions of hair follicles. These findings were consistent with dissecting folliculitis of the scalp. The patient was treated with oral antibiotics, intralesional corticosteroid, and high-potency topical corticosteroid, with partial inflammatory improvement and stabilization. This case highlights the value of clinicotrichoscopic-pathologic correlation in early dissecting cellulitis and raises the possibility that repeated procedural microtrauma, barrier disruption, follicular injury, or secondary bacterial colonization may act as a local trigger in a predisposed patient. The long-term exogenous testosterone exposure should be interpreted as a possible modifying factor rather than proof of causality.

## Introduction

Dissecting cellulitis of the scalp, also called perifolliculitis capitis abscedens et suffodiens or dissecting folliculitis, is a rare chronic inflammatory follicular occlusion disorder characterized by painful nodules, abscesses, draining sinus tracts, and progressive scarring alopecia [1,2]. It is considered part of the follicular occlusion spectrum and shares pathogenic overlap with hidradenitis suppurativa, acne conglobata, and pilonidal disease [1,2]. Pathophysiologically, follicular occlusion may lead to follicular dilation and rupture, followed by an intense neutrophilic, granulomatous, and mixed inflammatory response that can progressively destroy follicular units and result in cicatricial alopecia.

Early recognition is clinically relevant because timely control of recurrent deep inflammation may reduce permanent follicular loss and scarring. Although advanced disease is usually clinically evident, early presentations may be subtle and can be mistaken for non-scarring alopecias, focal folliculitis, traumatic alopecia, or progression of androgenetic alopecia.

Trichoscopy can aid early recognition by identifying follicular and perifollicular inflammatory changes before extensive sinus formation or irreversible scarring becomes clinically dominant [3-7]. In early or atypical disease, correlation between clinical morphology, trichoscopic findings, and histopathology is particularly valuable. The present case describes histopathologically supported early dissecting cellulitis in a patient with androgenetic alopecia after repeated scalp microinfusion therapy, with long-term exogenous testosterone use as an additional potential modifying factor.

## Case presentation

A 34-year-old male with androgenetic alopecia was receiving oral minoxidil 5 mg daily and had completed six-monthly sessions of scalp microinfusion of medication into the skin. The patient had a history of hypogonadism and had used exogenous testosterone for approximately two years. The microinfusion protocol included minoxidil 0.5%, finasteride 0.05%, growth factors, copper peptide, vitamins, D-panthenol, biotin, L-arginine, chromium, and copper. The technique was performed using a Cheyenne Unlimited 4 dermograph fitted with a 2710MG cartridge, corresponding to a 27-needle magnum configuration with 0.30 mm diameter needles, operating at 6.1 V, with the needle depth set to 1.2 mm. The last session was performed in January 2026.

**Figure 1:**
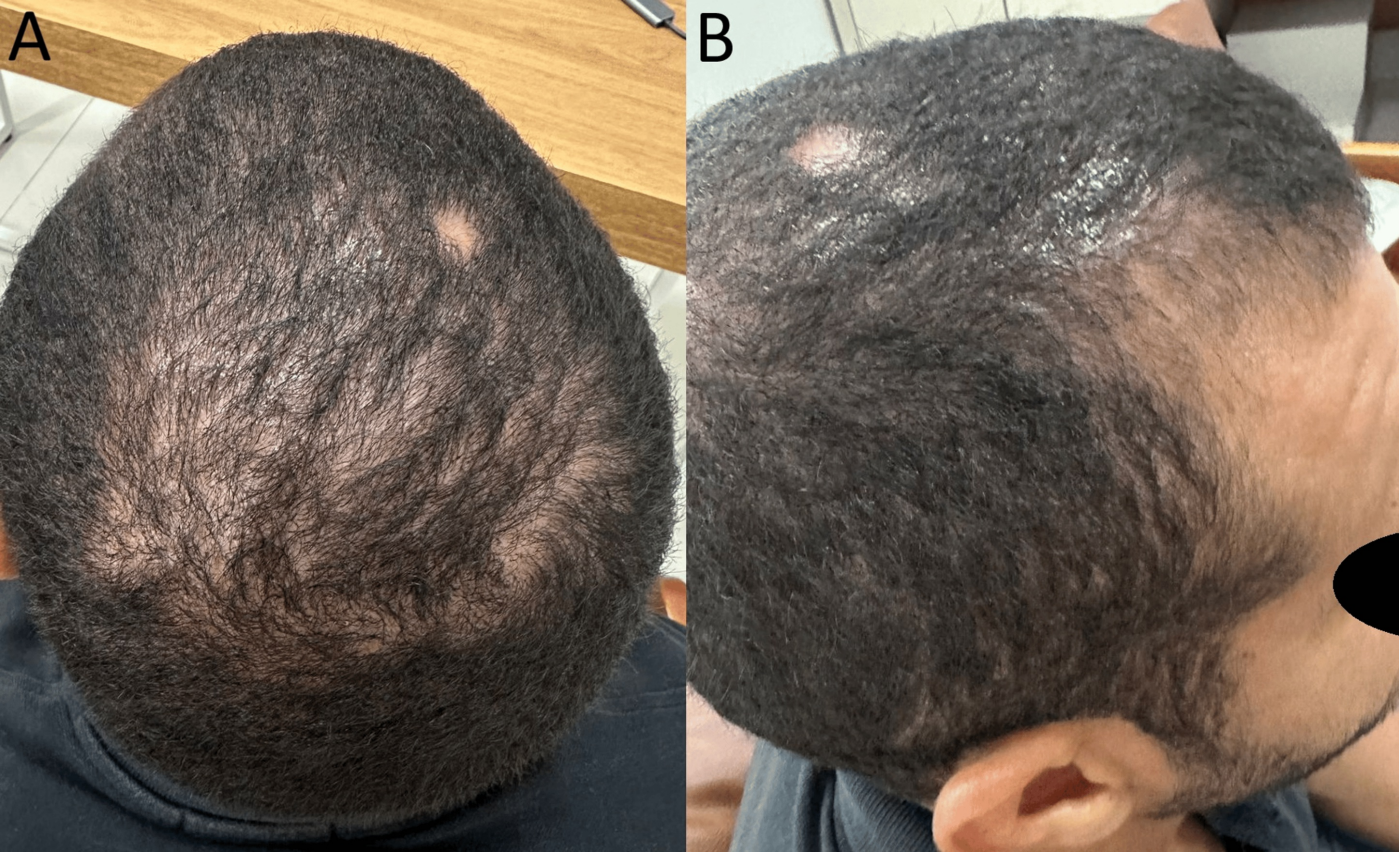
Clinical presentation of early dissecting cellulitis of the scalp in a patient with androgenetic alopecia (A) Vertex view showing diffuse androgenetic alopecia with reduced hair density and focal inflammatory alopecic plaques/nodules. (B) Lateral view showing an erythematous alopecic nodule on the frontoparietal scalp, without evident purulent drainage or draining sinus tracts. Identifying facial features were obscured.

Approximately 45 days after the final session, the patient developed a small inflammatory point on the scalp that progressively enlarged. A second lesion subsequently appeared in the vertex hair whorl region approximately 30 days after the first lesion. The lesions were associated with frequent pruritus, tenderness to touch, and occasional pain. At presentation, the first lesion measured approximately 1 cm in diameter, and the second approximately 0.4 cm. The patient denied previous similar episodes. Clinical examination revealed a tender erythematous alopecic nodule in the right frontoparietal scalp and a second erythematous papular lesion in the vertex hair whorl region, with localized hair rarefaction and no evident purulent drainage. Diffuse fronto-vertex rarefaction compatible with concomitant androgenetic alopecia was also present (Figure 1).

**Figure 2:**
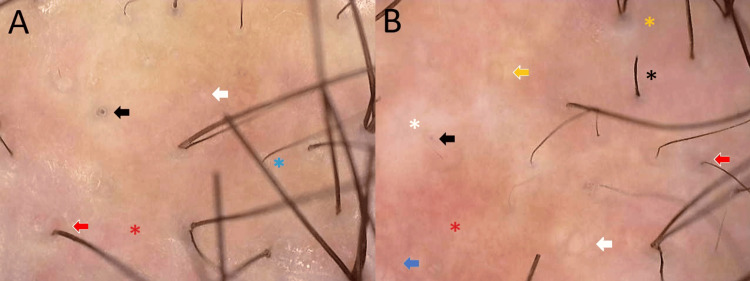
Trichoscopic findings of early dissecting cellulitis of the scalp Trichoscopic images at 70× magnification showing inflammatory and follicular changes in the affected scalp area. (A and B) Black arrows indicate black dots, white arrows indicate white dots, yellow arrow indicates yellow dots with a three-dimensional soap-bubble-like appearance, blue arrows indicate fine arborizing vessels, red arrows indicate perifollicular erythema, red asterisks indicate interfollicular erythema, white asterisk indicates an amorphous whitish area, black asterisk indicates a broken hair, yellow asterisk indicates a perifollicular pustule, and blue asterisk indicates perifollicular scaling.

Trichoscopy showed diffuse perifollicular erythema, interfollicular erythema, reduced follicular density in the involved area, predominance of single-hair follicular units, black dots, yellow dots with a three-dimensional soap-bubble-like appearance, broken hairs, dystrophic hairs, perifollicular pustule, white dots, white structureless areas, fine arborizing vessels, subtle perifollicular scaling, and mild hair shaft diameter diversity compatible with background androgenetic alopecia. Classic signs of alopecia areata, including typical exclamation mark hairs and classic yellow dots, were not observed. There were no convincing trichoscopic features of trichotillomania. The absence of exuberant pustules and draining sinus tracts, together with partial preservation of follicular openings, suggested an early inflammatory stage (Figure 2).

Laboratory testing performed two months before the final treatment session is summarized in Table 1. These findings were interpreted in the clinical context of exogenous testosterone use and dyslipidemia, both of which were relevant to therapeutic planning, particularly regarding retinoid risk-benefit considerations.

**Table 1:** Patient laboratory profile, including lipid, hematimetric, and hormonal parameters Laboratory findings showing hypercholesterolemia with markedly elevated LDL cholesterol, mild elevation of hemoglobin and hematocrit, and total and calculated free testosterone levels near or above the upper reference limits for adult males. Reference intervals may vary according to analytical method, patient age, laboratory standards, and the formula used to calculate free testosterone. HDL, high-density lipoprotein; LDL, low-density lipoprotein.

Parameter	Patient value	Reference range/clinical target	Interpretation
Total cholesterol	257 mg/dL	Desirable: <190 mg/dL	High
LDL cholesterol	200 mg/dL	Optimal: <100 mg/dL; ≥190 mg/dL: very high	Very high
HDL cholesterol	41 mg/dL	Low: <40 mg/dL; best/protective: ≥60 mg/dL	Borderline-low/below optimal
Hemoglobin	18.2 g/dL	Male: 13.8-17.2 g/dL	High
Hematocrit	51.70%	Male: 40.7%-50.3%	High/mildly elevated
Total testosterone	931 ng/dL	Adult male: 20-49 years, 249-836 ng/dL	Mildly elevated/above the reference range
Calculated free testosterone	285.56 pg/mL	Adult male: 20-49 years, 55.11-178.53 pg/mL	Elevated/above the reference range

**Figure 3:**
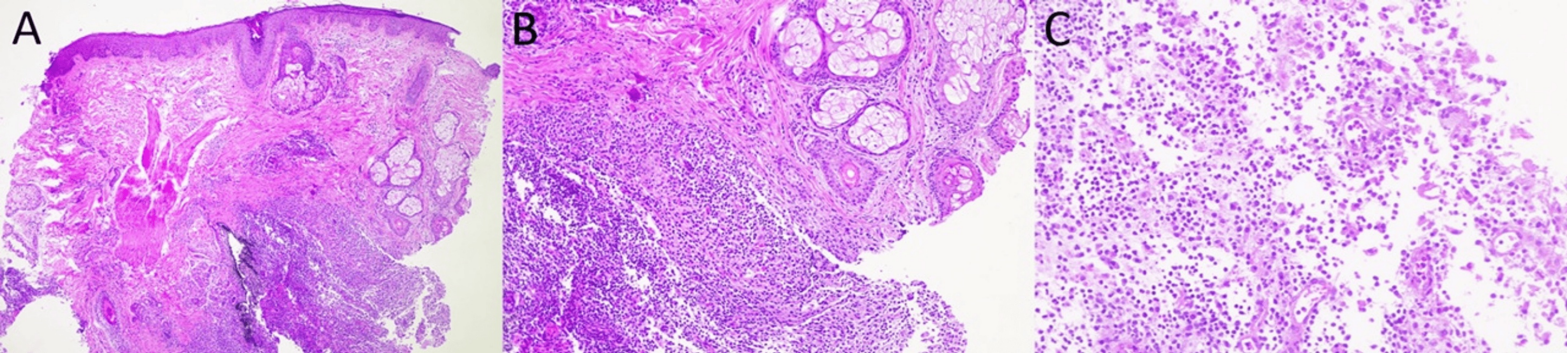
Histopathological findings of early dissecting cellulitis of the scalp Hematoxylin- and eosin-stained sections of the scalp biopsy. (A) Low-power view showing epidermis, dermis, adnexal structures, and a dense deep inflammatory infiltrate extending around follicular/adnexal structures and into deeper tissue planes, compatible with an early suppurative and granulomatous follicular process. Original magnification ×40. (B) Intermediate-power view showing perifollicular/adnexal inflammation, granulation tissue, and dense mixed inflammatory infiltrate adjacent to follicular and sebaceous structures. Original magnification ×100. (C) High-power view showing mixed leukocytic inflammation composed predominantly of neutrophils with lymphocytes, histiocytes/macrophages, plasma cells, and foreign body-type multinucleated giant cells. Original magnification ×400.

Scalp skin biopsies were obtained. Histopathological findings were obtained from the official pathology report; representative hematoxylin- and eosin-stained sections demonstrated fragments of scalp tissue with a region of the hypodermis occupied by granulation tissue and an intense mixed leukocytic infiltration composed of lymphocytes, plasma cells, neutrophils, macrophages, and foreign body-type multinucleated giant cells. In some areas, the inflammatory process involved the bulbar regions of hair follicles and perifollicular/adnexal structures and was associated with follicular miniaturization (Figure 3). Periodic acid-Schiff staining was negative for fungal structures. Bacterial colonization was identified in the infundibular and isthmic portions of hair follicles.

The availability of representative histopathological images further supports the diagnosis by illustrating the depth and composition of the inflammatory process. The low- and intermediate-power images (Figures 3A, 3B) demonstrate perifollicular/adnexal involvement and deep mixed inflammation, while the high-power image (Figure 3C) highlights the suppurative and granulomatous components, including foreign body-type multinucleated giant cells. These findings are compatible with early dissecting folliculitis/cellulitis of the scalp and help distinguish the process from isolated androgenetic alopecia, alopecia areata, dermatophyte infection, or primary traumatic alopecia.

The final diagnosis was early dissecting folliculitis/cellulitis of the scalp in a patient with androgenetic alopecia. The differential diagnosis included alopecia areata, focal bacterial folliculitis, dermatophyte infection, traumatic alopecia, trichotillomania, and other early cicatricial alopecias. Treatment was initiated with oral sulfamethoxazole-trimethoprim, 800 mg/160 mg twice daily for 14 days, followed by a dose reduction to 400 mg/80 mg twice daily for an additional 14 days. Two intralesional triamcinolone acetonide injections at a concentration of 2.5 mg/mL were administered at a 21-day interval. Clobetasol propionate 0.05% topical solution was also prescribed twice daily for 21 days, followed by once-daily application for an additional 21 days. Topical minoxidil 5% solution twice daily was associated with concomitant androgenetic alopecia management. A bacterial culture with an antibiogram was obtained; final results showed no bacterial growth. The patient showed partial reduction of inflammatory signs and clinical stabilization, without progression to evident purulent drainage, abscess formation, or draining sinus tracts. Biologic therapy, including tumor necrosis factor-alpha inhibitors, was reserved as a possible future option if the disease became refractory or progressive.

## Discussion

This case illustrates an early form of dissecting cellulitis in which clinical, trichoscopic, and histopathological correlation was necessary to avoid misclassification as progression of androgenetic alopecia or as a non-scarring inflammatory alopecia. The coexistence of patterned hair loss can mask early inflammatory nodules, particularly when focal rarefaction occurs in the frontal and vertex regions. Trichoscopic studies have shown that dissecting cellulitis can present with heterogeneous findings, including yellow dots, black dots, broken hairs, dystrophic hairs, perifollicular erythema, loss or reduction of follicular openings, and white structureless areas depending on disease activity and chronicity [3-7]. In the present case, the key early warning features were new-onset tender and pruritic inflammatory alopecic nodules, perifollicular and interfollicular erythema, perifollicular pustule, black dots, broken hairs, white structureless areas, partial preservation of follicular openings, and absence of classic trichoscopic features of alopecia areata or trichotillomania.

The histopathological findings in this case were particularly supportive. The combination of deep mixed inflammation, hypodermal granulation tissue, foreign body-type multinucleated giant cells, follicular involvement, negative fungal staining, and follicular bacterial colonization favored dissecting folliculitis over alopecia areata, isolated androgenetic alopecia, dermatophyte infection, or primary traumatic alopecia. The partial preservation of follicular openings and absence of extensive draining sinus tracts supported the interpretation of an early stage rather than advanced fibrosing disease.

The temporal relationship with repeated scalp microinfusion therapy deserves cautious interpretation. Microneedling and related pen-type procedures are generally considered minimally invasive and relatively safe, but adverse effects, including erythema, pain, edema, irritation, infection, and rare granulomatous reactions, have been described [8]. In the present case, repeated puncture-based treatment of the scalp to a depth of 1.2 mm may have produced local microtrauma, transient barrier disruption, follicular injury, or conditions favoring secondary bacterial colonization. These mechanisms are biologically plausible but do not establish causality. The correct interpretation is therefore a temporal association with a possible procedure-related trigger in a predisposed patient.

This procedural hypothesis is consistent with reports of dissecting cellulitis after hair restoration surgery and hair transplantation, in which follicular trauma, follicular unit manipulation, and local inflammatory responses have been proposed as possible contributors [9,10]. Unlike those reports, the present case followed repeated microinfusion therapy rather than follicular unit extraction or transplantation. This distinction may be clinically relevant because microinfusion and microneedling-based scalp therapies are increasingly used for hair disorders, including androgenetic alopecia. Clinicians should monitor for inflammatory nodules, pustules, pain, drainage, or focal alopecic plaques after such procedures, especially in patients with additional risk modifiers.

Long-term exogenous testosterone use is another relevant variable. Hidradenitis suppurativa and related follicular occlusion disorders may be influenced by hormonal and androgenic pathways, although direct causality in an individual patient is difficult to prove [11]. In this case, testosterone exposure had been present for approximately two years, making it less likely to be the sole acute trigger. However, it may have contributed to a permissive follicular occlusion milieu through androgen-sensitive pilosebaceous biology, sebaceous activity, or inflammatory modulation. The supraphysiologic total and free testosterone levels strengthen the rationale for mentioning testosterone as a possible modifying factor while avoiding causal overstatement.

Therapeutic evidence for dissecting cellulitis remains limited, and management is often stepwise and individualized. Antibiotics, intralesional or topical corticosteroids, oral retinoids, biologics, small-molecule inhibitors, laser approaches, and surgery have all been reported, depending on severity, activity, scarring, and refractoriness [12-15]. In this patient, dyslipidemia made systemic retinoids less attractive initially, while the absence of extensive abscesses or sinus tracts favored a conservative anti-inflammatory and antimicrobial approach. Early recognition may be prognostically important because preventing recurrent deep inflammation may reduce irreversible follicular destruction and cicatricial alopecia.

This report has limitations. Causality between repeated microinfusion therapy, exogenous testosterone exposure, and dissecting cellulitis cannot be established from a single case. Follow-up was limited to eight weeks, and objective severity scoring or serial standardized trichoscopic measurements were not performed. Despite these limitations, the temporal association, clinical morphology, trichoscopic findings, and histopathological photomicrographs/report support the diagnosis of early dissecting cellulitis and justify clinical awareness after puncture-based scalp procedures.

## Conclusions

Early dissecting cellulitis of the scalp may be difficult to recognize in patients with concomitant androgenetic alopecia, particularly when inflammatory alopecic nodules occur in areas already affected by patterned thinning. This case supports the importance of clinicotrichoscopic-pathologic correlation for early diagnosis and timely treatment. The temporal association with repeated scalp microinfusion therapy raises the possibility of a local procedure-related trigger through microtrauma, follicular injury, barrier disruption, or secondary bacterial colonization, while long-term exogenous testosterone exposure may represent a modifying factor rather than a proven cause. Causality cannot be inferred from a single case, but clinicians performing puncture-based scalp procedures should monitor for early inflammatory nodules and consider dissecting cellulitis in the differential diagnosis.
